# Association between oncogenic human papillomavirus type 16 and Killian polyp

**DOI:** 10.1186/s13027-020-00342-3

**Published:** 2021-01-07

**Authors:** Lucia Oton-Gonzalez, John Charles Rotondo, Luca Cerritelli, Nicola Malagutti, Carmen Lanzillotti, Ilaria Bononi, Andrea Ciorba, Chiara Bianchini, Chiara Mazziotta, Monica De Mattei, Stefano Pelucchi, Mauro Tognon, Fernanda Martini

**Affiliations:** 1 Department of Medical Sciences, Laboratories of Cell Biology and Molecular Genetics, School of Medicine, University of Ferrara, 64/B, Fossato di Mortara Street, 44121 Ferrara, Italy; 2 Department of Biomedical Sciences and Specialistic Surgeries, ENT Section, University of Ferrara and University Hospital of Ferrara, 8, Aldo Moro Square, 44124 Cona, Italy

**Keywords:** Killian polyp, Human papillomavirus, Polyomavirus, Infection, Nasal polyps

## Abstract

**Background:**

Killian polyp (KP) is a benign lesion that arises from the maxillary sinus. The etiology of KP is unknown. The aim of this study was to investigate the potential involvement of human papilloma- (HPV) and polyoma-viruses (HPyV) infections in the onset of KP.

**Methods:**

DNA from antral (*n* = 14) and nasal (*n* = 14) KP fractions were analyzed for HPV and HPyV sequences, genotypes, viral DNA load and physical status along with expression of viral proteins and p16 cellular protein.

**Results:**

The oncogenic HPV16 was detected in 3/14 (21.4%) antral KPs, whilst nasal KPs tested HPV-negative (0/14). The mean HPV16 DNA load was 4.65 ± 2.64 copy/10^4^ cell. The whole HPV16 episomal genome was detected in one KP sample, whereas HPV16 DNA integration in two KPs. P16 mRNA level was lower in the KP sample carrying HPV16 episome than in KPs carrying integrated HPV16 and HPV- negative KPs (*p*< 0.001). None of the antral and nasal KP samples tested positive for HPyV DNA (0/28).

**Conclusions:**

A fraction of KP tested positive for the oncogenic HPV16. HPV16 detection in the KP antral portion may be consistent with HPV16 infection derived from the maxillary sinus. HPV16 DNA integration represents a novel finding. Altogether, these data improve our knowledge on the association between KP and HPV infection, whereas it indicates that the KP onset is heterogeneous.

## Introduction

Killian polyp (KP), or antrochoanal polyp, is a benign lesion of the upper respiratory tract arising from the maxillary anthrum, which may extend through the nasal cavity to the choana. KP represents about 5 and 33% of nasal polyps in adults and children, respectively [1, 2]. KP usually presents as unilateral pedunculated mass composed by an antral portion, which is usually cystic, and a nasal/choanal fraction, emerging through an enlarged maxillary accessory ostium [3].

The etiopathogenesis of KP is not known. Several studies have suggested that autoreactivity, allergies and/or chronic inflammation could be risk factors for KP onset [4–6]. KPs are indeed inflammatory polyps [7]. Schryver et al. questioned if autoreactivity contributed to the KP onset or it resulted from a chronic inflammation, and proposed to investigate other inflammation causes, such as viral infections [8]. In fact, KP recurrence after its incomplete surgical removal suggests that viral infections may play a role [3, 9, 10].

Different viruses are able to infect the oropharyngeal region, and play a role in various head and neck diseases [11, 12]. Specifically, human papillomaviruses (HPV) and polyomaviruses (HPyV) such as BKPyV, JCPyV and Merkel cell polyomavirus (MCPyV), are DNA viruses infecting the tonsillar tissues [13–16], and have been associated to the development of respiratory diseases as well as to head and neck cancer [17–20]. HPV and HPyV display similar biological behavior in infected target tissues. After infection of epithelial cells, HPV and HPyV may multiply and spread in different anatomical sites, or enter lifelong latent phase, whereby viral DNA is maintained at low copy number [21, 22]. In some instance, long term latency of the oncogenic HPV and HPyV types may result in viral DNA integration into the host cell genome, leading to cell transformation upon viral oncoprotein overexpression [21–25].

The association between HPV infection and KP has been poorly investigated, whereas studies on HPyV in KP are missing. HPV sequences have been found at different prevalence, ranging 0–54% [26–28]. Moreover, oncogenic HPV genotypes such as HPV16, have been found to be prevalent in KPs, raising the question if HPV may play role in cell transformation. One recent study focusing on tumor marker expression, such as p16 and viral oncoproteins, did not find any correlation between HPV DNA positivity and KP development, concluding that HPV latently infects KP [27]. However, HPV DNA load and physical status, which are two main hallmarks of latent or active infection, have not been assessed yet in KP [29].

Even though maxillary sinus viral infections are considered risk factors for KP, there is no evidence proving the KP etiopathogenesis from this infection. So far, studies focusing on the identification of viral infections have analyzed bulk KP tissues without diversifying between the antral and nasal components. This distinction would be particularly important to understand if viral infections may play a role in the KP onset. In fact, any viral sequences detected in the antral region might account for maxillary sinus infections, and therefore potentially involved in the onset of KP, while those in the nasal region might be due to nasopharyngeal infections after the KP formation, thus not relevant for KP onset.

The aim of this study was to investigate the potential involvement of HPV and HPyV infections in the onset of KP. To this purpose, tissue samples from KP were divided into antral and nasal parts, and analyzed separately for HPVs and HPyVs sequences, genotypes, DNA load and physical status (episomal vs integrated), and expression, along with expression levels of p16, which is a cell protein strictly associated to active HPV infection.

## Materials and methods

### Samples

Killian polyp (KP) tissue specimens were collected from 14 patients (Mean age ± SD; 44 ± 18 years) who underwent surgical removal at the Ear, Nose and Throat Unit, University Hospital of Ferrara (Italy). Inclusion criteria were unilateral polyp with histopathological diagnosis of KP and age between 18 and 80 yrs. Exclusion criteria were bilateral polyps not coincident with KP. Written informed consent was obtained from all patients. The study was conducted in accordance with the Declaration of Helsinki. The protocol was approved by the County Ethical Committee (ID:160986).

### Nucleic acids extraction

KP tissue samples (*n*=14) were divided into two portions: the antral (*n*=14) and the nasal portion (*n*=14). Samples (*n*=28) were incubated overnight with proteinase K at 56 °C to allow tissue digestion. Then, nucleic acids were simultaneously extracted from samples using the All Prep DNA/RNA extraction kit (Qiagen, Milan, Italy). DNA from KPs was isolated/purified together with a salmon sperm DNA (ssDNA) sample and a mock sample lacking DNA [30]. After purification, DNAs/RNAs were quantified spectrophotometrically (NanoDrop 2000, Thermo Scientific) [31]. DNA amplification suitability was evaluated by *β-globin* gene PCR [32]. DNA/RNAs were stored at − 80 °C until time of analysis.

### Detection of HPV and HPyV DNAs

KP tissue samples were tested for HPV and BKPyV, JCPyV and MCPyV DNA sequences, by quantitative PCR (qPCR). Fifty ng of human genomic DNA were used in 10 μl qPCR reactions. For HPV DNA detection the universal primers GP5+/GP6+ (Table 1) were used, as previously reported [33, 34]. These primers allow simultaneous amplification of several HPV types [35, 36], including those frequently detected in KP, such as HPV6/11/16/18 [27, 28]. QPCR reactions included 2x of the SsoAdvanced Universal SYBR Green Supermix, Bio-Rad (Hercules, CA, USA) and a final concentration of 0.5 μM for each GP5+/GP6+ primer. For HPyV DNA detection, specific primers for BKPyV, JCPyV and MCPyV were employed [25, 38, 39]. QPCR reactions included 2x of the TaqMan Universal Master Mix II, no UNG, Thermo Fisher Scientific (Waltham, MA, USA), and 1X of primers and probe assays (Table 1). Recombinant plasmids containing HPV16 genome and BKPyV, JCPyV, and MCPyV genomes were used as positive controls [25, 38, 39], whereas ssDNA and mock samples lacking of DNA, as negative controls of DNA extraction and PCR amplification. Each assay was run in triplicate.

**Table 1:** Primers used in qPCR to detect and quantify HPV, PyV DNA, viral, cellular genes

	Target	Primers names	Primers sequence (5′→ 3′)	Amplicon size (bp)	Annealing temp. (°C)	References
**DNA**						
Viral	HPV L1	GP5+	TTTGTTACTGTGGTAGATACTAC	139–145	48	Malagutti et al. 2020 [33]; Tognon et al. 2020 [34]; Rotondo et al. 2020a [35]
		GP6+	GAAAAATAAACTGTAAATCATATTC			
	HPV16 E2	E- HPV16 E2 F	AACGAAGTATCCTCTCCTGAAATTATTAG	82	60	Peitsaro, Johansson, e Syrjänen 2002 [37]
		E- HPV16 E2 R	CCAAGGCGACGGCTTTG			
		E Probe 16E2PRO	[ROX] CACCCCGCCGCGACCCATA [BHQ2]			
	HPV16 E6	I+E- HPV16 E6 F	GAGAACTGCAATGTTTCAGGACC	81	60	
		I+E- HPV16 E6 R	TGTATAGTTGTTTGCAGCTCTGTGC			
		I+E Probe 16E6PRO	[6FAM] CAGGAGCGACCCAGAAAGTTACCACAGTT [BHQ1]			
	MCPyV	RQ MCPyV_LT.1F	CCACAGCCAGAGCTCTTCCT			Tagliapietra et al. 2020 [38]
		RQ MCPyV_LT.1R	TGGTGGTCTCCTCTCTGCTACTG			
		RQ MCPyV_LT Probe	[6FAM] TCCTTCTCAGCGTCCCAGGCTTCA [MGB]			
	JCPyV	Assay_JCyV	AI1RWNE			Tagliapietra et al. 2019 [39]
	BKPyV	Assay_BKyV	AI20UTM			
Host	β-Globin	β-Globin F	TGGGTTTCTGATAGGCACTGACT	152	56	Contini et al. 2018 [32]
		β-Globin R	AACAGCATCAGGAGTGGACAGAT			
**RNA**						
Viral	HPV16 E2	HPV16 E2 F	AACGAAGTATCCTCTCCTGAAATTATTAG	82	60	Peitsaro, Johansson, e Syrjänen 2002 [37]
		HPV16 E2 R	CCAAGGCGACGGCTTTG			
	HPV16 E6	HPV16 E6 F	GAGAACTGCAATGTTTCAGGACC	81	60	
		HPV16 E6 R	TGTATAGTTGTTTGCAGCTCTGTGC			
	HPV16 E5	16-E5 FWD	CGTCCGCTGCTTTTGTCTGTGTCTACATAC	89	60	Weyn et al. 2011 [40]
		16-E5 REV	CACCTAAACGCAGAGGCTGCTGTTATCCAC			
	HPV16 E7	E7 FWD	AGGAGGATGAAATAGATGGTCCAG	112	60	Pett et al. 2006 [41]
		E7 REV	CTTTGTACGCACAACCGAAGC			
Host	P16INK4A	p16 ink4a FWD	CCAACGCACCGAATAGTTACG	58	60	Marcoux et al. 2013 [42]
		p16 ink4a REV	GCGCTGCCCATCATCATG			
	GAPDH	GAPDH F	GAAGGTGAAGGTCGGAGTC	226	60	Xiao et al. 2011 [43]
		GAPDH R	GAAGATGGTGATGGGATTTC			

### HPV DNA load, genotype and physical status analyses

HPV DNA load was quantified by qPCR assay using the GP5+/GP6+ primers and a 10-fold dilutions standard curve, from 10^8^ to 10^2^ copies, of recombinant plasmids. HPV DNA load values were reported as viral copies per human cell equivalents (viral copy/cell). Samples were normalized vs. HPV16-positive SiHa cell line, which contains one HPV16 copy/cell. Human *β-globin* gene was used to determine the human cell equivalents of each sample [32]. HPV genotype was determined by differential melting temperature (T_m_), adding a high resolution melting (HRM) step, from 65 °C to 95 °C (ramping 0.1 °C every 0.03 s), to the qPCR analysis, as done before for detection of the HPV16 and HPV18 genotypes [44]. HPV6/11/16/18 plasmids were used as positive controls. HPV DNA physical status was investigated using the E2/E6 ratio by qPCR, as previously described (Table 1) [37]. Briefly, 50 ng of template DNA were analyzed in 10 μl multiplex PCR reactions, 2x TaqMan Universal Master Mix II, no UNG, Thermo Fisher Scientific (Waltham, MA, USA); 0.3 μM of each HPV16 E2 primer; 0.5 μM of each HPV16 E6 primer; and 0.1 μM of each E2 and E6 probe. E2/E6 ratio equal to 1 indicated episomal form, less or more than 1 mixed forms, i.e. episomal and integrated, whereas no E2 DNA detection indicated full integration. Each assay was run in triplicate.

### Rolling circle amplification (RCA) assay

The episomal viral DNAs were detected by rolling circle amplification (RCA) assay using the TempliPhi™ 100 Amplification Kit (GE Healthcare, Chicago, USA) [45], and in accordance with manufacturer’s instructions. Briefly, reactions were prepared with 25 ng of genomic DNA and 175 μM of dNTP mix (Thermo Scientific, Massachusetts, USA). The specificity of the RCA products was assessed by DNA restriction enzyme digestion in a final volume of 10 μL (Thermo Scientific, Massachusetts, USA). RCA and digested RCA products were visualized onto a 0.8% agarose gel. Positive and negative controls were used in the RCA assay.

### Gene expression analysis

Total RNA was retrotranscribed using the Improm II (Promega, Wisconsin, USA) reverse transcription system [46]. cDNAs were analyzed for the expression of HPV16 *E2*, *E6*, *E7* and *E5* genes and *p16* cellular gene (Table 1) [37, 40–42]. Briefly, 50 ng of cDNA were used in 10 μl reaction, 2x of the SsoAdvanced Universal SYBR Green Supermix, Bio-Rad (Hercules, CA, USA) and a final concentration of 0.5 μM for each primer [47]. *GAPDH* gene was employed as control for the gene expression analysis [43]. SiHa cell line was used as positive control for HPV gene expression and mock sample as negative control. Each assay was run in triplicate.

### Statistical analyses

Statistical analyses were performed using the GraphPad Prism for Windows (version 6.0, GraphPad, California, USA) [48, 49]. For mRNA, fold change was calculated by the 2^-ΔΔCt^ method and represented in Log_2_ scale, using HPV-negative samples as controls [31]. One-way analysis of variance was used to compare fold-change among samples [50]. *P* values less than 0.05 were considered statistically significant (*p*< 0.05) [51].

## Results

### Prevalence of HPV and HPyV sequences

DNAs isolated from KP tissue samples (*n*=28) represented by antral (*n*=14) and nasal (*n*=14) portions were tested for viral DNA sequences of HPV and HPyVs. The qPCR analyses showed that 3/14 (21.4%) of the antral KP tissues were positive for HPV DNA (Table 2). None of the nasal KP samples (*n*=14) tested positive for HPV DNA (0/14; 0%) (Table 2). KP tissue samples analyzed for BKPyV, JCPyV and MCPyV DNA sequences gave negative results in both antral (*n*=14) and nasal (*n*=14) portions (0/14; 0%) (Table 2).

**Table 2:** Prevalence of HPV and HPyV in antral and nasal KP tissues

Tissue sample	Number of positive samples/samples analyzed (%)			
	HPV	MCPyV	JCPyV	BKPyV
**Antral KP**	3/14 (21.4)	0/14 (0)	0/14 (0)	0/14 (0)
**Nasal KP**	0/14 (0)	0/14 (0)	0/14 (0)	0/14 (0)

### HPV DNA load, genotyping, and physical status analyses

**Fig. 1:**
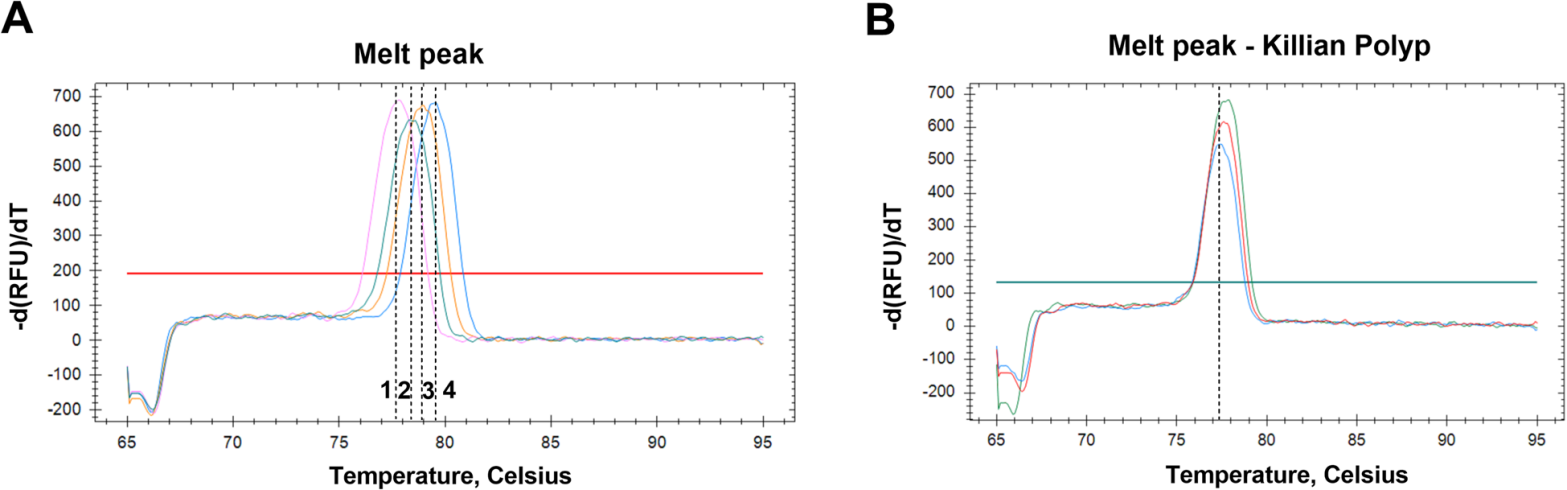
HPV differential melt peaks. **a** Melting temperature (Tm) for; 1) pUC19_HPV16; 2) pUC19_HPV11; 3) pUC19_HPV6 and 4) pUC19_HPV18. **b** Tm for KP samples, corresponding to that of pUC19_HPV16

HPV DNA load was determined by comparison to the HPV plasmid standard curve in qPCR assay. The mean viral DNA load in HPV-positive antral KPs (*n*=3) was 4.65±2.64 copy/10^4^ cell. In detail, in the three HPV-positive antral KP samples, the viral DNA load was 8.32 copy/10^4^ cell, 3.43 copy/10^4^ cell, and 2.21 copy/10^4^ cell. HPV genotype analyses were carried out by HRM qPCR assay. Firstly, the optimal T_m_ range for discriminating HPV6/11/16/18 types from GP5+/GP6+ amplicons was identified, which was between 75.4–79.5±0.2 °C (Fig. 1a). HPV genotype analyses were carried out by comparing qPCR T_m_ with the positive controls. Results indicated that the three HPV-positive antral KP samples carried the HPV type 16 (3/3; 100%) (Fig. 1b).

HPV16 DNA physical status was assessed by E2/E6 ratio in the three HPV16-positive antral KP samples. The E2/E6 ratio was 1.01 in one sample (1/3; 33.3%) indicating presence of HPV16 in the episomal form. In the two other samples only the E6 sequence was found (2/3; 66.6%), indicating that HPV16 was integrated into the host cell genome.

### HPV physical status validation by RCA

**Fig. 2:**
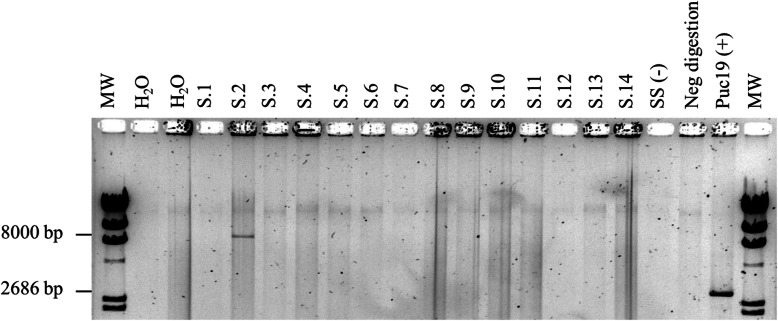
Rolling circle amplification assay performed on antral KP DNAs. MW: Molecular Weight. Negative controls: H_2_O, Salmon Sperm DNA (SS), Neg digestion (H_2_O). KP DNAs 1–14

Antral KP DNAs (*n*=14) were investigated by RCA for validating the HPV DNA episomal physical status. Successfully amplification was obtained only in the KP sample detected with E2/E6 ratio of 1.01, that was predictable of the episomal form. The molecular weight for the positive band corresponding to approximately 8000 bp was consistent of the HPV genome (Fig. 2, lane S2). Digestion with Bam HI enzyme, which cuts once into HPV16 genome, further confirmed the positivity for HPV16.

### Gene expression analysis

**Fig. 3:**
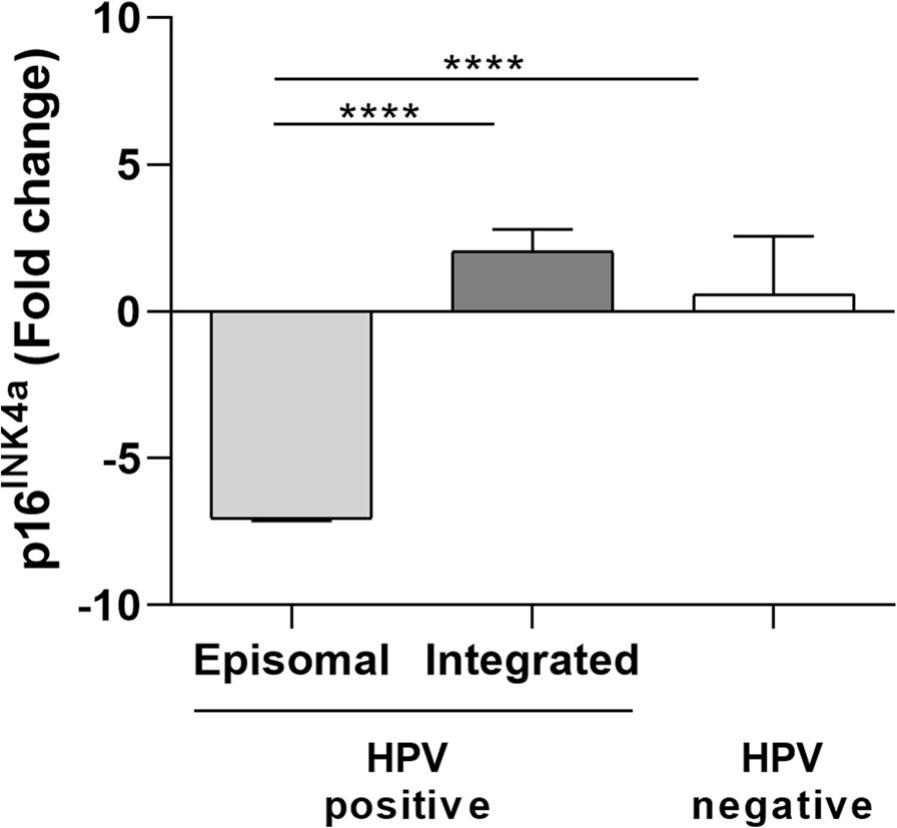
*p16* mRNA expression. KPs (*n*=14) were stratified according to HPV positivity/negativity. HPV-positive were further divided in episomal (*n*=1) and integrated (*n*=2). ****p< 0.0001

Viral gene expression was studied for the HPV-positive antral KP samples (*n*=3). No expression for HPV16 *E2*, *E5*, *E6*, *E7* genes was detectable in any of the samples analyzed, indicating either that HPV16 is not transcriptionally active in KP or that viral mRNA levels were too low to be detected under qPCR conditions. To gain insight into this topic, p16, which is considered a surrogate marker of active HPV infection, was analyzed for mRNA expression in the three antral HPV-positive KP samples, containing the episomal HPV16 (*n*=1) and the integrated HPV16 (*n*=2). Results indicated that *p16* mRNA level was 8.01-fold lower in HPV16-episome KP sample than in HPV16-integrated KP samples and 7.05-fold lower in HPV16-episome KP sample than in HPV-negative samples (*p*< 0.0001, Fig. 3). Although not statistically significant, *p16* expression was slightly higher in HPV16-integrated KP samples compared to HPV-negative samples (*p*> 0.05, Fig. 3).

## Discussion

The etiopathogenesis of KP is still not completely understood. Viral infections have been suggested to be involved in KP onset [52, 53]. Herein, with the aim to verify the putative role of the viral infections, HPV and HPyV were investigated in KP samples. Independent analyses of the antral and nasal region were useful in understanding whether the KP infections depended on the maxillary sinus or the nasal cavity.

HPV sequences were detected in 21% of the antral KP samples, while none of the nasal samples tested positive for HPV. This result indicates that the KP antral region is target of HPV infection and suggests a possible link between maxillary sinus infections and KP development. In term of prevalence, our data are in agreement with previous studies reporting HPV rates ranging from 0 to 54% in KP samples, although the new methodological approach used herein does not allow our and previous data to be compared adequately [26, 27, 54–60].

HPV genotypes have been investigated in two previous studies reporting HPV16 to be frequently detected at higher rate, 61.9 and 85.72%, respectively, than HPV11, 14.3 and 14.28%, respectively [27, 28]. In this study HPV16 was the only viral genotype detected in KP. These results are of interest as HPV16 is the high risk oncogenic type involved in development of different tumors [24, 61–63], including head and neck cancer [64, 65].

HPV viral load and physical status are indicative of active or latent infection in the infected tissues [29, 66]. For the first time, DNA load and physical status was investigated in HPV-positive KP samples. The viral DNA load was lower than 1 copy/cell, which is consistent with latent or persistent infection occurring in normal tissues [67, 68]. When HPV physical status was analyzed a heterogeneous trend was found among the HPV-positive KP samples. One sample carried HPV16 in episomal form, which was confirmed amplifying the whole HPV genome by RCA assay. Instead, two KP samples showed the HPV16 DNA in integrated form. This is an interesting finding because high risk HPV integration into the host cell genome is a common event preceding cell transformation [69]. On the other hand, HPV integration occurs up to 42.8% of normal tissues, as previously reported in HPV-positive normal cervical samples [70]. Regard KP, evidences proving its neoplastic transformation do not exist, although some cases mimicking malignant transformation have been reported [71]. Nevertheless, HPV carcinogenesis in KP, if any, could be difficult to be assessed, since KPs are removed early after presentation, whereas HPV transformation process occurs in long lasting time, needing many years to be detected. Altogether, our data indicate that HPV16 is present at low DNA load in both episomal and integrated form, consistent with latent/persistent infection in the antral KP. Nevertheless, the detection of the oncogenic HPV16 combined with its DNA integration in the KP is intriguing. Further studies are needed to assess the HPV DNA integration in KP over the time.

HPV mRNA expressions occur during active viral infection. Accordingly, in this study, viral expression of *E2*, *E5*, *E6* and *E7* sequences was not detected in the HPV-positive KP samples. Although *E6/E7* expression in HPV-positive KP samples carrying viral DNA integration would be expected, HPV latency in normal and pathological tissues presenting viral DNA integration is also common [72]. Some other explanations may account for lack of viral expression. For instance, KPs are covered by ciliated cylindrical epithelium, which may be not permissive for HPV *E6/E7* gene expression [7, 67]. Also, it is possible that viral mRNA levels were too low to be detected under our qPCR conditions. Further studies with more sensitive assays may clarify this matter [46].

Since no HPV transcriptional activity was found, the surrogate marker of active HPV infection, the *p16*, was studied in correlation to infection. During HPV infection the viral E7 protein inactivates pRb tumor suppressor protein leading to p16 overexpression [73]. In this study, no difference between HPV16-positive KP samples carrying integrated viral DNA and HPV-negative KPs was observed (*p*> 0.05), although a slightly higher *p16* mRNA level was found in HPV16-positive KPs. Likely, the small samples size used in the study did not allow statistical significance to be reached. In contrast, the KP sample carrying episomal HPV DNA showed stronger *p16* down-expression compared to HPV-positive and HPV-negative KP samples (*p*< 0.001). Mutations at the *p16* coding gene may explain its down-expression [74, 75]. Alternatively, methylation at *p16* promoter may silence the gene leading to decrease expression, as previously shown in HPV-positive samples carrying HPV in episomal form [76].

Finally, HPyV DNA sequences were analyzed in KP. HPyVs have been found associated to different diseases, including cancer and polyposis [77]. Specifically, JCPyV has been studied in correlation to colon polyposis [78], whereas BKPyV has been investigated in the prostate and colon cancer onset [79]. MCPyV is the main cause of the Merkel cell carcinoma, a rare but very aggressive non-melanoma skin cancer [25]. Moreover, MCPyV is considered to be a part of the skin microbiota, and viral DNA sequences have been found in nasal swabs, blood, chorionic villi, eyebrows and adrenal glands [38, 77, 80]. In this study, HPyV sequences were not found neither in antral nor nasal KPs, thus excluding their role in KP formation.

## Conclusions

The present study investigated HPV and HPyV as potential pathogenic risk factors in KP. While no implication was found for HPyV, a fraction of KPs showed positivity for HPV16. New information on HPV DNA load and physical status in KPs were also provided. Specifically, HPV16-positive KPs presented viral DNA at low load and in episomal or integrated form. The reduced sample size employed in this pilot study could be considered a limitation, and further studies in a larger samples size are needed, especially for clarifying the oncogenic HPV16 integration into the KPs. Of note, KP samples were divided in antral and nasal portions, whereas HPV sequences were found only in the antral region, providing a possible explanation for polyp formation from sinus maxillary infections. We suggest that a HPV latent infection of the maxillary sinus might be responsible for its recurrence, after KP surgical removal, highlighting the importance of complete surgical removal of the HPV-positive pathological tissue to prevent further recurrences.

## Data Availability

Data and material will be available upon request to the corresponding author.

## Abbreviations

KP: Killian polyp
HPV: Human papillomavirus
HPyV: Polyomaviruses
RCA: Rolling circle amplifications
MCPyV: Merkel cell polyomavirus
ssDNA: Salmon sperm DNA
qPCR: quantitative PCR
